# Dietary pro-oxidant score (POS) and cardio-metabolic panel among obese individuals: a cross-sectional study

**DOI:** 10.1186/s12902-023-01395-2

**Published:** 2023-07-10

**Authors:** Negin Nikrad, Amir Shakarami, Zahra Rahimi, Raheleh Janghorbanian -Poodeh, Mahdieh Abbasalizad Farhangi, Babak Hosseini, Faria Jafarzadeh

**Affiliations:** 1 Tabriz Health Services Management Research Center, Tabriz University of Medical Sciences, Tabriz, Iran; 2 Department of Cardiovascular Medicine, Assistant Professor of Cardiology, Lorestan University of Medical Sciences, Khorramabad, Iran; 3 Teaching Experimental Sciences Group, Teachers Training Center, Pardis Bahonar Faculty of Farhangian University, Isfahan, Iran; 4 Coronary Angiography Group, Heart Department of Chamran Sub-Speciality Heart Hospital, Isfahan University of Medical Sciences, Isfahan, Iran; 5 Department of Community Nutrition, Faculty of Nutrition, Tabriz University of Medical Sciences, Tabriz, Iran; 6 Department of Surgery, School of Medicine, Laparoscopy Research Center, Shiraz University of Medical Sciences, Shiraz, Iran; 7 Department of Internal Medicine, School of Medicine, North Khorasan University of Medical Sciences, Bojnourd, Iran

**Keywords:** Dietary pro-oxidant score, Obesity, Metabolic parameters, Blood pressure, Lipid profile

## Abstract

**Background:**

Oxidative stress is a disturbance in the natural balance between oxidative and anti-oxidative processes, which is the major effective factor in cardiovascular disorders and metabolic syndrome (MetS), due to the role of pro-oxidants in inducing oxidative stress, and as a result, the occurrence and exacerbation of components of metabolic syndrome and cardiovascular risk factors, this cross-sectional study was conducted with the aim of investigating the relationship between the status of dietary pro-oxidants score (POS) and metabolic parameters including serum lipids, glycemic markers and blood pressure among obese adults.

**Methods:**

338 individuals with obesity (BMI ≥ 30 kg/m ^2^), aged between 20 and 50 years were recruited in the present cross-sectional study. A validated food frequency questionnaire (FFQ) was used to determine the dietary pro-oxidant score (POS). Analysis of variance (ANOVA) with *Tukey’s* post-hoc comparisons after adjustment for confounders and multivariable logistic regression analysis were performed to determine the association of cardiometabolic risk factors among the tertiles of POS.

**Results:**

Participants with higher POS had lower levels of body mass index (BMI), weight and waist circumference (WC). There were no significant associations between metabolic parameters including glycemic markers and lipid profile in one-way ANOVA and multivariate multinomial logistic regression models.

**Conclusions:**

The findings of this study revealed that greater dietary pro-oxidant intake might be associated with lower BMI, body weight, and WC in Iranian obese individuals. Further studies with interventional or longitudinal approaches will help to better elucidate the causality of the observed associations.

## Introduction

Oxidative stress (OS) is a multifaceted, complicated process that develops from an imbalance between reactive oxygen species generated by pro-oxidants and antioxidant defense [1]. Numerous studies have demonstrated that cellular pathways’ high oxidative stress is a major contributor to cardiovascular disorders and manifestations of the metabolic syndrome MetS [2–4]. Multiple metabolic abnormalities, including obesity, type 2 diabetes (T2D), and the consequences of T2D known as the MetS, are caused by imbalances in the energy and redox potential of cells, these imbalances are triggered by the disruption of important biological reactions [5]. MetS is characterized as a pathophysiological association and combination of cardiometabolic risk factors that are known to increase a person’s risk of CVD and T2DM [6]. According to studies, MetS affects over 30% of the world’s population, making it a serious worldwide public health problem [7], in Iran, MetS presently affects 30.4% of the population and is increasing significantly [8]. Pathophysiology of MetS is a highly complicated and unclear. The concept that disruption of the normal balance between oxidative and anti-oxidative processes, resulting in redox state in the cell and tissues, may play a significant role in its manifestations is supported by a number of studies [5, 9]. A cross-sectional study on 113 elderly subjects indicated a strong relationship between the accumulation of Mets components and the aggravation of oxidative stress [10]. According to the research conducted so far, there is absolutely no doubt that oxidative stress and MetS are related [11], so many studies are focused on preventing (OS) in MetS. Diet could provide more precursors for endogenous lipid peroxidation and induce oxidative stress biomarkers by dietary components such as polyunsaturated fatty acids particularly ω-3 fatty acids with a high double bond index and heme iron [12]. Through the Fenton reaction, iron may produce reactive oxygen species (ROS), which results in oxidative stress and greater rates of lipid peroxidation [13]. Syrovatka P et al. [14] shown a substantial association between ferritin and obesity parameters and metabolic syndrome in healthy males, suggesting that higher body iron storage may cause reduced insulin sensitivity through increased oxidative stress [14]. Another dietary pro-oxidant is PUFAs, which are more vulnerable to oxidation and the production of LDL-oxidized blood due to their high level of unsaturation [15], highly unsaturated n-3 PUFA supplements have been shown to enhance oxidative stress in both humans and animals according to research [16–19]. Despite the fact that it is well recognized that saturated fatty acids (SFAs) in general encourage abdominal obesity, dyslipidemia, insulin resistance, systemic inflammation and impaired glucose tolerance [20–22]. SFAs overexposure has been shown to enhance the production of pro-inflammatory cytokines, disrupt insulin signaling, and drive apoptosis marked by both endoplasmic reticulum (ER) deficits and oxidative stress in a variety of cell types in vitro [23–26]. Leptin, an adipocyte-derived hormone that is increased in obese people and may cause oxidative stress, could be the physiological factor behind the association between obesity and oxidative stress [27]. Considering the role of pro-oxidants in oxidative stress and as a result the increases in the number of MetS components and cardio-metabolic risk factors, as well as limited research focusing only on pro-oxidant factors, this present study aimed to evaluate the association between dietary triggers of oxidative stress and cardio metabolic risk factors including blood pressure, lipid profile, glycemic and insulin related factors among apparently metabolically healthy subjects with obesity.

## Method & materials

### Study population

In the present cross-sectional study, 338 randomly chosen volunteers who were both male and female with obesity (BMI ≥ 30 kg/m^2^) and between the ages of 20 and 50 were chosen from previous research [28, 29] which have been done in the last four years from 2019 to 2022. The study excludes women who are pregnant, breast feeding, or postmenopausal, individuals who have had gastric bypass surgery or other weight loss operations, cancer, liver or kidney problems, cardiovascular diseases, diabetes mellitus, or cancer, and subjects who take any medications or dietary supplements that could affect weight. Meanwhile, the study protocol was approved by the ethics committee of Iran’s Tabriz University of Medical Sciences after all participants read and signed an informed consent form (Registration number: IR.TBZME-D.REC.1400.454).

### Sociodemographic data

We gathered demographic information through questionnaires and interviews. The socioeconomic status (SES) score was estimated using the data gathered on educational attainment, employment status, home ownership, and family size. Individuals reported their greatest degree of education, and we used education as a categorical variable. This variable was rated on a scale of 0 to 5 points as follows: illiterate: 0, less than diploma: 1, associate degree: 2, bachelor: 3, master: 4, and higher: 5. The employment status of female participants was divided into five groups (housewife, employee, student, self-employed, and others), while the occupational status of male participants was divided into the following categories: unemployed: 1, worker, farmer and rancher: 2, others: 3, employee: 4, and self-employed: 5. Individuals were categorized as belonging to families of 3 and under 3 (score 1), 4–5 (score 2), and over 6 (score3) persons. In addition, if they didn’t own a home, they got a score of 1, and if they did, they got a score of 2. Depending on the respondents’ total SES score, which varied from 1 to 15, the individuals were then categorized into three categories. Using the Persian version of the DASS-21[30], the frequency of depression, anxiety, and stress-related symptoms during the preceding weeks was evaluated. Age, marital status, educational level, nursing experience, work unit, shift work, and work hours per week are the seven items on this questionnaire that evaluate a subject’s sociodemographic features. A maximum score of three and a minimum score of zero are assigned to the questions on a Likert scale. On each scale, the score may be between 0 and 21. A visual analogue scale (VAS) was used to assess the state of the appetite in the morning following fasting state (VAS)[31]. To illustrate the two extremes of the VAS, the phrases “I’m not at all hungry” and “I have not been so hungry” were inserted at the opposite ends of a 100-mm line. This questionnaire asked about past and projected food intake as well as questions regarding hunger, satiety, fullness, cravings for sweet, salty, and fatty meals. A shortened version of the international physical activity questionnaire (IPAQ) was used to estimate the participants’ level of physical activity [32–34].

### Dietary assessments and calculation of dietary pro-oxidant score (POS)

We used a validated, semi-quantitative food frequency questionnaire (FFQ) that was adapted for the Iranian population to gather data on dietary consumption. The Iranian household manual’s recommendations for portion sizes, cooking yields, and dietary food amounts were used to ask participants to keep records of all the foods and beverages they consumed on a daily, weekly, monthly, or yearly basis. Every food item’s reported frequency was modified to daily intake and converted to grams. In this study, three dietary components known to have a pro-oxidant effect were selected including iron and saturated fatty acids (SFA) and polyunsaturated fatty acids (PUFA) [35–37]. The total amount of consumption of all three factors was calculated and they were categorized into tertiles by reverse scoring. The higher POS denotes the lower dietary pro-oxidant intake and was more favorable.

### Anthropometric assessments

A wall stadiometer with a sensitivity of 0.5 cm Seca scale (Seca Co., Hamburg, Germany) was used for measuring the participants’ height while individuals were asked to take off their shoes for height measurement. Weight of participants was measured, without extra clothes, by using Seca scale (Seca Co., Hamburg, Germany) with 0.1 kg precision. The bioelectrical impedance analysis (BIA) method was employed by Tanita, BC-418 MA (Tokyo, Japan) to estimate body composition which measures the body fat percentage, fat mass (FM), fat free mass (FFM), and predicted muscle mass. When exhaling, the distance between the iliac crest and the lowest rib was measured using a tape measure to the closest 0.1 cm.

### Measurement of blood biomarkers and blood pressure assessments

All measurements were taken after at least 12 h of overnight fasting. A trained physician used a standard mercury sphygmomanometer to measure the subject’s systolic and diastolic blood pressure on the right arm after 10 to 15 min of rest and sitting state. The average of two measured blood pressure was recorded for each participant. All individuals provided 10 ml of venous blood for sampling, which was centrifuged at 4500 rpm for 10 min to separate the serum and plasma samples. A commercial kit was used to analyze the serum levels of total cholesterol, triglycerides, high-density lipoprotein cholesterol, and fasting blood glucose (Pars Azmoon, Tehran, Iran). Additionally, the Friedewald equation was used to quantify the amount of low-density lipoprotein cholesterol [38]. Enzyme-linked immunosorbent assay (ELISA) kit was used to determine serum concentrations of insulin (Bioassay Technology Laboratory, Shanghai Korean Biotech, Shanghai City, China). Fasting insulin (IU/ml) + fasting glucose (mmol/l)/22.5 was used to calculate the homeostatic model assessment for insulin resistance (HOMA-IR), and the quantitative insulin sensitivity check index (QUICKI) was estimated as 1/[log fasting insulin (U/mL) + log glucose (mmol/L)].

### Statistical analysis

The Statistical Package for Social Sciences (version 21.0; SPSS Inc, Chicago IL) was used to perform the statistical analysis, with a significance level of P < 0.05. For categorical variables, data were reported as frequency (%), and for continuous variables, as mean (standard deviation). The Chi-square test and one-way analysis of variance (ANOVA) were used, respectively, to assess the differences in discrete and continuous variables across different tertiles of POS. In addition, three multivariable-adjusted and unadjusted models of multinomial logistic regression were used to estimate odds ratios (ORs) and 95% confidence intervals (CIs) for test the presence of possible association of cardiometabolic risk factors among the POS tertiles.

## Results

The present cross-sectional study was conducted among 338 obese participants (mean BMI of 32.62 kg/ m^2^ and mean age of 40.78 years old) which includes 41% male. Table 1 shows the general characteristics and biochemical parameters of the participants across quartiles of POS. Older subjects scored higher on the prooxidant score, indicating a direct relationship between age and dietary prooxidant intake (P = 0.03). According to the results of this study, a higher POS that indicates lower intake of pro-oxidants was accompanied by lower weight (P = 0.004), lower BMI levels (P = 0.001). WC was also lower in higher tertiles of POS than in lower tertile (P = 0.02). The significant level of association between BMI, weight and WC and POS remained even after adjusting for confounding factors (P < 0.001). No significant difference in other demographic variables such as SES score, DASS, appetite, physical activity was found among POS tertiles (P > 0.05). Tables 2 and 3 show the comparison of dietary intake of POS components, energy, macronutrients, and food groups in different tertiles of POS. A statistically meaningful lower intake of POS components (including iron, SFA and PUFA)(Table 2), fruit, meat group, energy, fat and MUFA was observed in the highest tertiles of POS (P < 0.05),while percentage of carbohydrate intake was higher in higher tertiles of POS (P < 0.001)(Table 3). Table 4 represents the odds ratios (ORs) and 95% confidence intervals (CIs) of the cardiometabolic risk factors across different tertiles of POS after adjustment for potential confounders in three models of crude, adjusted for age and sex and adjusted for age, sex, BMI, SES and dietary energy intake. Subjects in the second tertile of POS had a reduced risk of higher SBP levels (OR = 0.968, P = 0.04), and a higher risk of increased DBP levels (OR = 1.042, P = 0.05) compared with those with the lower PCOS tertiles in the crude and age, sex adjusted models. However, after adjustment for age and gender, BMI, SES and dietary energy intake (3rd model), these associations lost their significance.

**Table 1:** General demographic characteristics of study participants by tertiles of dietary pro-oxidants status

Variable	Tertiles of POS						
	1st ^(N=113)^		2nd ^(N=113)^		3rd ^(N=112)^		P* value
	Mean	SD	Mean	SD	Mean	SD	
Age (y)	39.60	8.96	39.77	8.77	42.52	9.62	0.03*
Gender (% Male)	61.9	38.05	53.1	50.12	56.3	49.83	0.39**
Weight	94.58	14.39	93.19	13.50	88.50	14.79	0.004* < 0.001***
Height	168.06	9.96	167.87	10.16	167.76	9.68	0.97*
BMI (kg/m^2^)	33.55	5.22	33.12	4.37	31.36	4.58	0.001* < 0.001***
WC (cm)	108.52	10.43	106.56	8.84	104.99	9.21	0.02* < 0.001***
FM (%)	34.33	9.32	33.87	9.98	32.97	7.65	0.72*
FFM (%)	62.98	11.82	61.99	13.40	61.56	11.81	0.81*
WHR	0.94	0.069	0.93	0.09	0.93	0.07	0.31*
SES score	9.84	2.34	10.02	2.66	10.04	2.57	0.88*
DASS	20.60	12.12	20.43	11.37	19.14	11.08	0.77*
Appetite	35.30	9.14	32.73	8.93	32.24	8.40	0.11*
PA (min/week)	2096.37	2885.95	2598.97	2915.99	1660.30	2542.20	0.29*
BMR (Kcal)	7843.13	1828.32	7906.82	1557.79	7806.73	1381.97	0.94*
SBP (mmHg)	121.56	19.08	122.21	15.01	124.50	14.11	0.36***
DBP (mmHg)	79.51	13.23	82.13	11.31	83.32	10.29	0.05***
FBS (mg/dl)	92.50	16.70	95.01	26.39	90.85	12.21	0.27***
TC (mg/dl)	185.57	33.96	196.64	35.49	192.95	40.35	0.07***
TG (mg/dl)	147.11	99.33	148.92	81.52	156.01	98.68	0.75***
HDL (mg/dl)	43.20	9.43	44.39	9.56	43.04	9.60	0.51***
LDL (mg/dl)	117.80	29.26	126.76	32.33	125.98	33.96	0.07***
Insulin (mIU/l)	15.52	11.39	17.47	17.39	15.69	11.91	0.60***
HOMA-IR	3.55	2.56	4.20	4.06	3.60	3.14	0.37***
QUICKI	0.33	0.04	0.32	0.03	0.33	0.04	0.08***

POS, pro-oxidants status; BMI, Body mass index; WC, Waist Circumference; FM, Fat Mass; FFM, Fat Free Mass; WHR, waist-to-hip ratio; BMR, Basal Metabolic Rate; SES, socio-economic status; DASS, depression anxiety stress scale; PA, physical activity; SBP, Systolic Blood Pressure; DBP, Diastolic Blood Pressure; TC, Total Cholesterol; TG, Triglyceride; HDL-C, High Density Lipoprotein Cholesterol; LDL-C, Low Density Lipoprotein Cholesterol; HOMA-IR, Homeostatic Model Assessment for Insulin Resistance; QUICKI, Quantitative Insulin sensitivity Check Index; all data are mean (± SD) except gender, that is presented as the number and percent of males in each group. P* values derived from One-Way ANOVA with Tukey’s post-hoc comparisons. ** P values derived from chi-squared test. P*** values derived from One-Way ANOVA with Tukey’s post-hoc comparisons after adjustment for confounders (age, gender, BMI, PA and kcal)

**Table 2:** Dietary intakes of POS components according to tertiles of dietary pro-oxidants status

Variable	Tertiles of POS						
	1st ^(N=113)^		2nd ^(N=113)^		3rd ^(N=112)^		P* value
	Mean	SD	Mean	SD	Mean	SD	
Iron (mg/d)	32.51	13.07	22.29	5.31	16.52	4.10	**< 0.001**
SFA (g/d)	43.10	16.52	27.61	6.58	17.31	4.24	**< 0.001**
PUFA (g/d)	34.92	15.15	20.14	5.49	12.69	3.37	**< 0.001**

POS, pro-oxidants status, SFA, saturated fatty acids, PUFA, polyunsaturated fatty acids. P-values are derived from one-way ANOVA test

**Table 3:** Food groups intake of study participants by tertiles of dietary and non-dietary pro-oxidants status

Variable			**Tertiles of POS**					
	**1st** ^**(N=113)**^		2nd ^**(N=113)**^		3rd ^(N=112)^		P* value	P** value
	Mean	SD	Mean	SD	Mean	SD		
Fruits (g/d)	5.11	3.61	3.96	3.06	3.06	1.79	**0.001**	**0.01**
Vegetables (g/d)	4.97	2.64	3.66	1.81	2.50	1.18	**< 0.001**	0.59
Dairy (g/d)	2.68	1.51	1.89	1.14	1.45	0.74	**< 0.001**	0.30
Beans	1.02	0.99	0.63	0.40	0.50	0.31	**< 0.001**	0.72
Fish group	0.38	0.56	0.26	0.27	0.28	0.31	0.21	0.50
Meat group	2.17	1.68	1.23	0.85	0.92	0.67	**< 0.001**	**0.001**
Poultry group	0.88	0.72	0.72	0.61	0.66	0.57	0.13	0.73
Grains (g/d)	18.41	7.48	13.23	5.48	9.59	3.81	**< 0.001**	0.09
Energy (kcal/d)	4162.21	982.01	2837.38	471.88	2053.83	386.74	**< 0.001**	**< 0.001**
CHO (%)	55.50	6.89	58.04	6.65	61.46	5.78	**< 0.001**	**< 0.001**
Protein (%)	12.69	2.05	12.86	1.85	13.71	1.91	**0.01**	0.45
Fat (%)	34.31	6.99	31.82	6.41	27.59	5.54	**< 0.001**	**< 0.001**
Cholesterol (mg/d)	401.62	171.30	288.82	238.20	198.64	113.36	**< 0.001**	0.076
MUFA (g/d)	49.96	17.28	30.29	6.12	19.53	4.79	**< 0.001**	**0.043**

CHO, carbohydrate, MUFA, mono-unsaturated fatty acids. All data are mean (± SD). P* values derived from unadjusted ANCOVA P** values derived from ANCOVA after adjustment for confounders (age, gender, BMI, PA and energy intake)

**Table 4:** Biochemical variables of study participants by tertiles of dietary and non-dietary pro-oxidants status

Variable		Tertiles of POS				
		1st ^(N=113)^	2nd ^(N=113)^		3rd ^(N=112)^	
			OR(CI)	P-value	OR(CI)	P-value
SBP (mmHg)	Model I	**1** **REF**	0.968 (0.939–0.998)	**0.04**	0.989 (0.959–1.020)	0.47
	Model II		0.971(0.941–1.002)	0.07	0.989(0.959–1.020)	0.48
	Model III		0.988(0.922–1.059)	0.74	0.966(0.877–1.063)	0.47
DBP (mmHg)	Model I	**1** **REF**	1.042(1.000-1.086)	0.05	1.028(0.986–1.072)	0.19
	Model II		1.044(1.001–1.089)	**0.04**	1.025(0.983–1.069)	0.25
	Model III		1.023(0.944–1.108)	0.58	0.978(0.867–1.104)	0.72
FBS (mg/dl)	Model I	**1** **REF**	1.007(0.977–1.038)	0.65	0.982(0.950–1.015)	0.29
	Model II		1.016(0.984–1.049)	0.32	0.984(0.951–1.018)	0.34
	Model III		0.995(0.933–1.062)	0.88	1.015(0.880–1.172)	0.83
TC (mg/dl)	Model I	**1** **REF**	0.988(0.958–1.018)	0.43	0.999(0.968–1.030)	0.93
	Model II		0.986(0.953–1.021)	0.43	0.999(0.966–1.033)	0.95
	Model III		1.013(0.989–1.037)	0.30	1.020(0.988–1.053)	0.21
TG (mg/dl)	Model I	**1** **REF**	1.003(0.997–1.010)	0.35	1.003(0.996–1.009)	0.42
	Model II		1.004(0.997–1.012)	0.26	1.003(0.996–1.010)	0.43
	Model III		1.012(0.998–1.026)	0.10	1.019(0.999–1.039)	0.07
HDL (mg/dl)	Model I	**1** **REF**	1.025(0.981–1.071)	0.27	1.003(0.957–1.051)	0.90
	Model II		1.021(0.973–1.071)	0.40	0.998(0.950–1.048)	0.93
	Model III		0.990(0.918–1.068)	0.80	0.912(0.804–1.034)	0.15
LDL (mg/dl)	Model I	**1** **REF**	1.021(0.989–1.054)	0.20	1.010(0.978–1.043)	0.54
	Model II		1.024(0.988–1.061)	0.20	1.010(0.976–1.045)	0.57
	Model III		1.024(0.982–1.063)	0.21	1.012(0.979–1.049)	0.59
Insulin (mIU/l)	Model I	**1** **REF**	0.992(0.864–1.140)	0.91	0.937(0.808–1.087)	0.39
	Model II		1.023(0.887–1.180)	0.75	0.946(0.811–1.103)	0.48
	Model III		0.774(0.521–1.149)	0.20	1.388(0.634–3.043)	0.41
HOMA-IR	Model I	**1** **REF**	0.991(0.538–1.826)	0.98	1.345(0.704–2.572)	0.37
	Model II		0.885(0.471–1.664)	0.70	1.282(0.654–2.511)	0.47
	Model III		1.208(0.367–3.977)	0.76	0.192(0.014–2.728)	0.22
QUICKI	Model I	**1** **REF**	7.232E-6(2.144E-12-24.393)	0.12	1.271(4.132E-6-391102.367)	0.97
	Model II		2.517E-5(4.287E-12-147.813)	0.18	1.039(2.552E-6-422810.689)	0.99
	Model III		1.962E-19(1.479E-45-26021916.72)	0.16	166.243(1.036E-40-2.668E + 44)	0.92

SBP, Systolic Blood Pressure; DBP, Diastolic Blood Pressure; TC, Total Cholesterol; TG, Triglyceride; HDL-C, High Density Lipoprotein Cholesterol; LDL-C, Low Density Lipoprotein Cholesterol; HOMA-IR, Homeostatic Model Assessment for Insulin Resistance; QUICKI, Quantitative Insulin Sensitivity Check Index; OR, odds ratio; CI, confidence interval. The multivariate multinomial logistic regression was used for estimation of ORs and confidence interval (CI). Model I: crude, Model II: adjusted for age and sex, Model III: adjusted for age, BMI, sex, physical activity, SES and energy intake

## Discussion

**Fig. 1:**
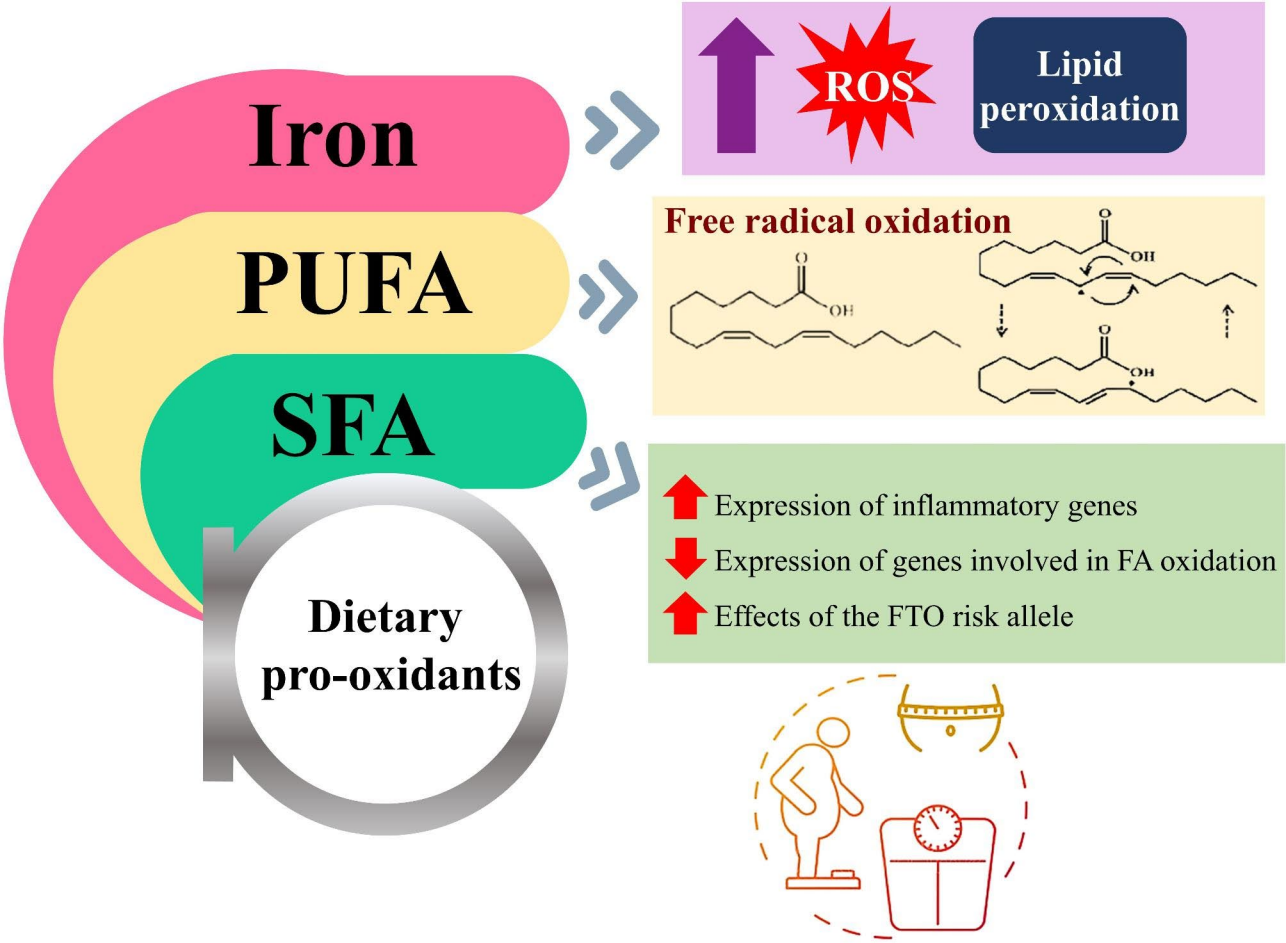
Graphic abstract of the possible mechanisms of dietary pro-oxidants in association of body weight, BMI and WC. Abbreviations: PUFA, polyunsaturated fatty acids; SFA, saturated fatty acids; ROS, reactive oxygen species; FA, fatty acid; FTO, fat mass and obesity-associated

To the best of our knowledge, no prior research has been conducted to explore the association between dietary pro-oxidants and cardiometabolic risk factors. In this cross-sectional research of obese adults, we evaluated dietary pro-oxidant consumption, indicated by pro-oxidant score (POS), and its relationship with cardiometabolic risk factors. As the primary finding of the present study, a better status of dietary pro-oxidants (determined by higher POS tertiles with a reduction in the consumption of dietary factors including iron, PUFA, and SFA that can induce pro-oxidant state) was associated with improved weight, BMI, and waist circumference (WC). Consumption of a high SFA diet led to an increase in the expression of inflammatory genes in adipose tissue and a decrease in the expression of genes involved in fatty acid -oxidation and the synthesis of triglycerides, according to a parallel controlled-feeding trial carried out in 20 people who were centrally overweight who are at risk of metabolic syndrome [39], this finding can explain the association of SFA as one of the pro-oxidant factors and obesity that we indicated. Furthermore, significant interactions between genetic risk score and total fat, SFA, and MUFA intake were discovered in 497 Asian Indian individuals, revealing that high SFA intake is significantly associated with larger WC than low SFA intake in individuals with high genetic risk, also low SFA intake was shown to be associated with smaller WC in individuals with higher genetic risk compared to those with lower genetic risk [40]. A study involving 2163 participants from the US population hypothesized that SFA may play a major role in modulating the effects of fat mass and obesity associated (FTO) polymorphisms that are associated with BMI/obesity, results indicated that high-SFA intake instead of total fat intake could be more associated in increasing the effects of the FTO risk allele on BMI [41]. In addition, the intake of SFA, MUFA, and PUFA is positively correlated with the risk of obesity and higher BMI, according to epidemiological cross-sectional research [42–44]. While PUFA-rich diets, particularly n-3 PUFA, are thought to be useful in preventing several metabolic disorders [45–47], the other side of the coin is that PUFAs are vulnerable to free radical oxidation[48], which makes them turn into a pro-oxidant agent. Results of a cross-sectional study with 895 individuals demonstrate strong associations between PUFAs and ROS generation in young and middle-aged groups in multiple regression models, suggesting that PUFAs may enhance oxidative stress and have deleterious consequences for the body [49, 50]. However, the reduction of systolic and diastolic blood pressure by PUFA supplementation has been demonstrated in treated hypertensive subjects [51]. In addition, an increase in the content of these fatty acids in cell membranes is linked to a decrease in blood pressure caused by omega-3 PUFAs, which is likely to depend on the composition of the cell membrane at birth, which in turn may be influenced by dietary patterns and even genetic factors [52]. Results of a meta-analysis of 70 randomized controlled trials (RCTs) which investigates the effect of omega-3 PUFAs including eicosapentaenoic acid + docosahexaenoic acid (EPA + DHA) on BP showed that both hypertensive and normotensive patients had statistically significant BP reductions brought on by omega-3 PUFA [53]. This effect of PUFAs on blood pressure could justify the effect of increasing diastolic blood pressure in second tertile of POS that we observed in multivariate multinomial logistic regression (Table 4). Excess iron levels are harmful and can produce reactive oxygen species that cause lipid peroxidation and DNA damage [54–58] as well as increased prevalence of metabolic syndrome its components [59, 60]. A meta-analysis of fourteen observational studies indicated that the dietary iron level has significant positive association with MetS [61]. In a cross-sectional research of 1567 Japanese individuals with type-2 diabetes, Ferreira ED et al.[62] found that regardless of macronutrient and fiber intake, participants in the highest quartile of iron intake had a significantly higher risk of obesity in the 30- to 54-year-old age group. Meanwhile, studies have shown that serum ferritin level has a positive correlation with WC, BMI [63–65]. Hence, the mentioned findings could be consistent with our findings regarding the significant relationship between pro-oxidant factors and weight, BMI, and WC (Table 1). The summary of the mechanisms of these three pro-oxidant factors involved in the regulation of obesity is given in Fig. 1. According to results of our research, there is no correlation between tertiles of POS and SBP, DBP, FBS TC, TG, HDL-C, LDL-C, insulin, HOMA-IR, or QUICKI. Noruzi et al. also reported no significant relationship between components of the MetS and combined pro- and antioxidant exposure status, indicated by oxidative balance score, except for increased WC and DBP among 847 Iranian participants [36, 66]. In contrast, Abbasiana et al. [67] revealed a significantly meaningful correlation between the oxidative stress indicators such as total antioxidant capacity and malondialdehyde and number of Mets components among 167 adult participants.

This study was not without limitations. At first, the study’s cross-sectional design restricts the casual inference, longitudinal investigations are required to clarify the cause-effect relationships. Second, this study was conducted in apparently healthy people aged 20–50 in the two centers of the provinces of Iran, Tabriz and Tehran, so generalizing the results to people with metabolic disorders /other age groups / other parts of the country should be done with caution. Not only the FFQ was not originally designed to assess POS, but the use of questionnaires can increase recall errors and create errors in data collection related to consumption reports. It is also better in further studies to measure the levels of pro-oxidant and antioxidant enzymes such as NADPH oxidase, xanthine oxidase and advanced glycation end products (AGE), superoxide dismutase, catalase, etc. be taken. Nevertheless, the present study also had several strengths. According to the knowledge of authors, this is the first cross-sectional study to examine the association between dietary consumption of pro-oxidants and cardiometabolic risk factors. Also three models were used to modify the multivariate multinomial logistic regression for a large number of potential confounding variables, which increased the reliability of the findings.

## Conclusions

The results of the current study showed that lower intake of pro-oxidants is associated with reduced risk of general and central obesity. Nutritional clinical trainings with emphasis on following a diet with less content of dietary prooxidants can be effective in improving weight status and body mass index and waist circumference. Longitudinal or interventional studies are recommended to find the causal relationships between dietary pro-oxidants and metabolic risk factors.

## Data Availability

The datasets generated and/or analyzed during the current study are not publicly available due to privacy and ethical considerations, but can be available from the corresponding author on reasonable request.
